# Lysine Acetylation, Cancer Hallmarks and Emerging Onco-Therapeutic Opportunities

**DOI:** 10.3390/cancers14020346

**Published:** 2022-01-11

**Authors:** Meilan Hu, Fule He, Erik W. Thompson, Kostya (Ken) Ostrikov, Xiaofeng Dai

**Affiliations:** 1 Wuxi School of Medicine, Jiangnan University, Wuxi 214122, China; humeilan2022@163.com; 2 Department of Prevention and Health, Affiliated Hangzhou First People’s Hospital Zhejiang University School of Medicine, Hangzhou 310000, China; 3 First School of Clinical Medicine, Zhejiang Chinese Medical University, Hangzhou 310053, China; hefule@163.com; 4 School of Biomedical Sciences, Queensland University of Technology, Brisbane, QLD 4059, Australia; e2.thompson@qut.edu.au; 5 Translational Research Institute, Woolloongabba, QLD 4102, Australia; 6 Centre for Biomedical Technologies, School of Chemistry and Physics, Queensland University of Technology, Brisbane, QLD 4000, Australia; kostya.ostrikov@qut.edu.au

**Keywords:** lysine acetylation, cancer hallmarks, cold atmospheric plasma, cancer epigenomics, onco-therapeutics

## Abstract

**Simple Summary:**

Several histone deacetylase inhibitors have been approved by FDA for cancer treatment. Intensive efforts have been devoted to enhancing its anti-cancer efficacy by combining it with various other agents. Yet, no guideline is available to assist in the choice of candidate drugs for combination towards optimal solutions for different clinical problems. Thus, it is imperative to characterize the primary cancer hallmarks that lysine acetylation is associated with and gain knowledge on the key cancer features that each combinatorial onco-therapeutic modality targets to aid in the combinatorial onco-therapeutic design. Cold atmospheric plasma represents an emerging anti-cancer modality via manipulating cellular redox level and has been demonstrated to selectively target several cancer hallmarks. This review aims to delineate the intrinsic connections between lysine acetylation and cancer properties, and forecast opportunities histone deacetylase inhibitors may have when combined with cold atmospheric plasma as novel precision onco-therapies.

**Abstract:**

Acetylation, a reversible epigenetic process, is implicated in many critical cellular regulatory systems including transcriptional regulation, protein structure, activity, stability, and localization. Lysine acetylation is the most prevalent and intensively investigated among the diverse acetylation forms. Owing to the intrinsic connections of acetylation with cell metabolism, acetylation has been associated with metabolic disorders including cancers. Yet, relatively little has been reported on the features of acetylation against the cancer hallmarks, even though this knowledge may help identify appropriate therapeutic strategies or combinatorial modalities for the effective treatment and resolution of malignancies. By examining the available data related to the efficacy of lysine acetylation against tumor cells and elaborating the primary cancer hallmarks and the associated mechanisms to target the specific hallmarks, this review identifies the intrinsic connections between lysine acetylation and cancer hallmarks and proposes novel modalities that can be combined with HDAC inhibitors for cancer treatment with higher efficacy and minimum adverse effects.

## 1. Introduction

Acetylation is a metabolic process that plays critical roles in many cellular processes of human life and a chemical process that enzymatically modifies the acetyl group of a protein/peptide or messenger RNA (mRNA) [1,2]. Protein acetylation mainly occurs on lysine, serine, and threonine residues, with lysine acetylation being the dominant type given its wide cellular compartment distribution as well as prominent roles in chromatin remodeling, physiological metabolism, and other key features that are relevant to various diseases including cancers [3]. mRNA acetylation was discovered in 2018 and occurs at N4-acetylcytidine (ac4C) through the catalytic effect of the acetyltransferase NAT10. It was demonstrated to enhance protein translation in vitro and in vivo [4]. 

Protein acetylation was first proposed in 1964 where Allfrey et al. reported ε-amino acetylation of lysine residues in histone and clarified the roles of protein acetylation in reducing the inhibition of RNA synthesis and promoting gene transcription [5]. Yet, it was not until 1996 when Allis et al. reported the histone acetyltransferase A (HATA) catalytic activity of Gcn5p [6] and Schreiber et al. reported the histone deacetylase (HDAC) activity of Rpd3 [7] in the nucleus, that a significant advance was made in this field [1]. In 2006, protein acetylation was found to be prevalent in mitochondria, suggesting its existence in other cellular compartments besides the nucleus; Zhao et al. reported the regulatory role of mitochondria acetylation on acetyl-CoA synthetases (AceCSs) by identifying 388 acetylation sites among 195 proteins using a novel acetylation-specific antibody-based detection approach [8], and associating the mitochondria acetylation with cell metabolism [9]. In 2009, Mann et al. identified 3600 lysine acetylation sites in 1750 proteins and observed its prevalent involvement in many cellular events through conducting an in-depth analysis on the acetyl groups in cells using high-resolution mass spectrometry [10]. Nowadays, studies on acetylation have expanded from exploring the occurrence site and chemical mechanism to the translational implications of acetylation such as the diagnostic and therapeutic potential in human diseases such as cancer and metabolic disorders [10].

Focusing on lysine acetylation, this paper will briefly introduce the primary classification and basic features of lysine acetylation, associate lysine acetylation with cancer hallmarks [11] to facilitate translational studies in this field and present our insights to guide the future developments in the field with emphasis on the implications of lysine acetylation in human cancers and the development of promising therapeutic approaches. 

## 2. Lysine Acetylation Classification and Basic Features

Over 80% of human proteins bear N-terminal acetylation at the α-amino position of the first amino acid [12]. Acetylation of key lysine residues can occur enzymatically or non-enzymatically to affect its intermolecular interactions, functionalities, stability, and localization. Lysine acetylation had once been considered as a unique modification of histones but has recently been found residing in thousands of non-histone proteins located in almost every cellular compartment such as nucleus, mitochondria, and cytoplasm.

Lysine acetylation is a reversible epigenetic modification controlled by lysine acetyltransferases (KATs) and lysine deacetylases (KDACs, Sirtuin). As such, it represents an important epigenetic modulatory process of both histone and non-histone proteins. During lysine acetylation, an acetyl group is transferred from acetyl-coenzyme A (acetyl-CoA) to the ε-position of the lysine side chain of a protein, where the positive electrostatic charge of that specific loci is neutralized. Non-enzymatic acetylation also occurs, the level of which is determined by the counteracting effects of the ‘writer’ (acetyltransferases) and the ‘eraser’ (deacetylases). Acetylated lysine residues can be identified by the ‘reader’ proteins that harbor specific acetyl-lysine binding domains for function interpretation.

KATs from distinct families harbor homologous acetyl-CoA binding domains, the flanking regions of which determine their enzyme specificities [13]. The best characterized acetyl-CoA binding domain is the bromodomain. Almost all bromodomain-containing proteins are located in the nucleus to regulate chromatin structure and functions. Other acetyl-CoA binding domains include the PHD domain in proteins DPF3b, CHD4, and KAT6A, as well as the YEATS domain in YEATS2, ENL, AF9, TFIIF, and GAS41 [14,15]. While the bromodomain, PHD, and YEATS domains read acetylated lysine residues, the SET domain identifies non-acetylated lysine enriched regions of H3, KU70, p53, and FOXO1 [16]. 

**Figure 1:**
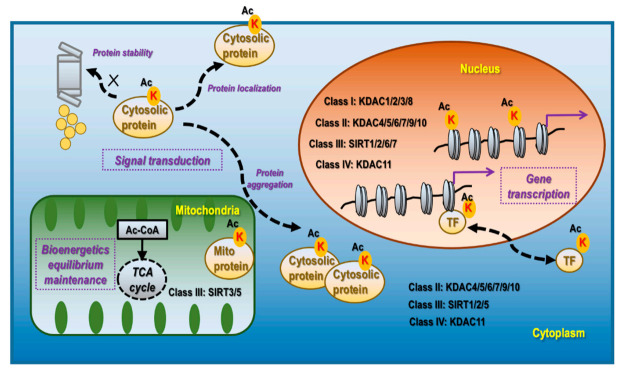
Cellular distribution, corresponding deacetylases, and primary functionalities of lysine acetylation. Lysine acetylation occurs in the nucleus, mitochondria, and cytoplasm. Nucleus lysine acetylation is deacetylated by class I deacetylases KDAC1/2/3/8, class II deacetylases KDAC4/5/6/7/9/10, class III deacetylases SIRT 1/2/6/7, Class IV deacetylase KDAC11; mitochondria lysine acetylation is deacetylated by class III deacetylases SIRT3/5; cytoplasm lysine acetylation is deacetylated by class II deacetylases KDAC4/5/6/7/9/10, class III deacetylase SIRT1/2/5, and class IV deacetylase KDAC11. Nucleus lysine acetylation can affect gene transcription via modulating chromatin structure if occurring on histones and regulate gene expression via controlling the localization, expression, and activity of TFs as well as the transcriptional machinery. Mitochondrial lysine acetylation can control the maintenance of bioenergetic equilibrium as acetyl-CoA is the substrate of acetylation and TCA cycle, and an important product of glycolysis. Cytoplasmic acetylation can affect signal transduction via modulating protein stability, localization, and aggregation, alone or together with other PTM programs.

KDACs and Sirtuin (SIRT) are mechanistically and structurally distinct deacetylases, which are classified into four categories. Class I, II, and IV deacetylases are Zn^2+^-dependent and include KDACs 1-11. While class I members (KDAC1/2/3/8) are localized primarily in the nucleus, class II (KDAC4/5/6/7/9/10) and class IV (KDAC11) deacetylases exist both in the nucleus and cytoplasm (Figure 1). Class III deacetylases comprise SIRTs1–7, which are distributed in the nucleus (SIRsT1/2/6/7), cytoplasm (SIRTs1/2/5), and mitochondria (SIRTs3/5) (Figure 1). Out of the seven Sirtuins in mammals, only SIRTs 1/2/3 present robust lysine deacetylase activity [17].

Acetylation in the nucleus is highly effective in inducing active cell division, including in tumor cells, and can occur on histones [18], transcription factors (TFs) [19], and basal transcription machinery [20]. The acetylation functionalities are associated with gene transcription regulation as summarized in Figure 1. The mechanism of lysine acetylation is related to the exposure of DNA to the transcription machinery by disrupting the electrostatic interactions between the phosphodiester backbones of DNA and lysine enriched nucleosomes [21]. Acetylation modulates transcription factor activities via inducing nuclear translocation, triggering protein stabilization, modifying molecular complex composition, and enhancing the specificity and selectivity of chromatin binding. For instance, acetylation of the TF STAT3 at K685 induces its homodimerization and nucleus translocation [22]; acetylation of the TF p53 at K120 and K382 prevents it from MDM2-mediated ubiquitination and degradation [23]. Besides TFs, many factors associated with the RNA polymerase II complex are acetylated. For example, the C-terminal domain of RNA polymerase II is acetylated in actively transcribed genes, the lack of which leads to polymerase pausing [20].

Acetylation in the mitochondria plays a fundamental role in maintaining the cellular equilibrium of bioenergetics that is enriched in metabolically active tissues such as the liver [24] and the heart [25] (Figure 1). Approximately one-third of mitochondrial proteins are acetylated, many of which harbor multiple acetylated lysines [8]. Acetylated mitochondrial proteins are involved in many functions relevant to cellular metabolism including the TCA cycle, oxidative phosphorylation, lipid β-oxidation, carbohydrate metabolism, nucleotide metabolism, amino acid metabolism, and the urea cycle [26]. For instance, decreased acetylation of two mitochondrial proteins PDHA1 and PDP1 suppresses their functionalities, which leads to altered glucose homeostasis and the Warburg effect [27]; SIRT3 regulates ATP synthase via deacetylating proteins involved in the mitochondrial respiratory chain complexes such as SDHA [28].

Acetylation in the cytoplasm contributes, alone or together with other post-translational modification (PTM) events, to the regulation of signal transduction via affecting protein stability, aggregation, and localization, which are associated with protein turnover, activity, cytoskeleton regulation, and cell migration [29] (Figure 1). Though relatively less studied, it is currently considered to be predominantly present in the liver, peri-renal, testis fat, neuron, and tissues with high levels of acetyl-CoA [17]. For instance, under the context of neuro-degeneration, α-tubulin acetylation at K40, occurring at the luminal side of microtubules, is a marker of protein stability [30]; acetylation of Tau at K280 promotes its aggregation while acetylation at K274 and K281 leads to its mis-localization, and acetylation at K174 slows its cellular turnover, which collectively contribute to cognitive impairment [31]; PD-L1 de-acetylation on K263 in the cytoplasmic domain by HDAC2 is a prerequisite of its nuclear translocation that determines the efficacy of anti-PD-1 immunotherapy [32].

## 3. Intimate Connections between Lysine Acetylation and Cancer Hallmarks

**Figure 2:**
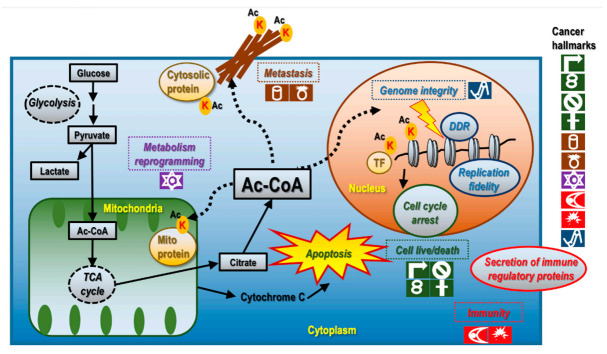
Associations between lysine acetylation and cancer hallmarks. Lysine acetylation dynamically interacts with cancer cell metabolism as acetyl-CoA is the acetyl-donor of acetylation, and acetylation could modulate cell metabolism if occurring on proteins affecting the expression of TFs or enzymes involved in cellular metabolism. Lysine acetylation, if residing in genes regulating core elements of adaptive or innate immune response, could lead to aberrant immune and inflammatory responses. Lysine acetylation is involved in genome integrity control as it could orchestrate the DDR machinery activities via modulating chromatin structure where many DDR events occur and could thus affect replication fidelity. Lysine acetylation could modulate cancer cell live/death and metastasis as it can occur on histone or non-histone proteins relevant to DDR, cell cycle progression and metastasis.

The established 10 cancer hallmarks, revised in 2011 [11], cover five cancer themes. In brief, ‘sustaining proliferative signaling’, ‘evading growth suppressors’, ‘resisting cell death’, ‘enabling replicative immortality’ are different causes leading to disordered cell life/death control; ‘inducing angiogenesis’ and ‘activating invasion and metastasis’ are relevant to cancer metastasis; ‘deregulating cellular energetics’ is associated with cancer metabolism; ‘avoiding immune destruction’ and ‘tumor-promoting inflammation’ are relevant to immunity; and ‘genome instability & mutation’ refers to DNA damage response (DDR) and genome instability. Lysine acetylation plays critical roles in enabling malignant cells with cancer hallmarks by being actively involved in modulating the levels and functionalities of genes and proteins with tumor promotive or suppressive roles and is associated with each of these cancer hallmarks (Figure 2). 

### 3.1. Acetylation and Cancer Cell Life/Death Control 

Histone acetylation is a prerequisite of chromatin de-condensation [33]. Histone acetylation could trigger cell cycle arrest and cell death, and histone deacetylase (HDAC) inhibitors have been proposed as a potential epigenetic onco-therapy [34]. For instance, H4 acetylation could induce G2/M cell cycle arrest in Raji cells by closing chromatin structures and decreasing the expression of target genes including *CDK1* and *cyclin B1* [35]. Quisinostat, an HDAC inhibitor, could trigger cell cycle arrest and apoptosis of lung cancer cells A549 via maintenance of H3 and H4 acetylation [36].

Acetylation of non-histone proteins, mostly relevant to DDR and cell cycle progression, could halt the cell cycle and induce cell death. For instance, sinomenine could arrest malignant glioma cells U87 and U251 at the G0/G1 stage and induce apoptosis via down-regulating SIRT1, which acetylates p53 [37] at probably K382/K373 [36]. Lamin B1 (LMNB1) acetylation at K134 could slow the G1/S cell cycle transition via inhibiting the recruitment of 53BP1 to damaged DNA that negatively regulates non-homologous end joining and halts cell cycle progression [38]. MORC2 acetylation at K767 could be activated by DNA-damaging agents and ionizing radiation; this down-regulates H3 phosphorylation at T11. These mechanisms lead to the suppressed expression of cell cycle genes *CDK1* and *cyclin B1* and sensitize breast cancer cells to the treatment of DNA-damaging chemotherapy and radiotherapy towards cell death [39]. 

### 3.2. Acetylation and Cancer Cell Metastasis 

Histone acetylation can both promote or suppress cancer metastasis. As an example of promoting tumor metastasis, *COL6A1* (commonly over-expressed in osteosarcoma) could be up-regulated by enhanced enrichment of H3K27 acetylation at the promoter region, which increases osteosarcoma lung metastasis via interacting with SOCS5 towards suppressed *STAT1* expression and converting normal fibroblasts to cancer-associated fibroblasts (CAFs) [18]. As an example of histone acetylation with tumor-suppressive roles, H3K56 acetylation at the promoter region of *EGFR*, *CTNNB1* (encoding β-catenin), and *CDH1* suppressed prostate cancer migration and invasion [40].

Non-histone acetylation can also modulate cancer metastasis in both directions depending on the role that the acetylated protein plays in cancer progression. Acetylation of the TF KLF5 at K369 in advanced prostate cancer leads to osteoclastogenesis and chemotherapy-resistant bone metastasis via activating CXCR4 and consequently SHH/IL6 paracrine signaling [19]. SPZ1 acetylation at K369 and K374 coupled with TWIST acetylation at K73 and K76 is required for SPZ1-TWIST1 complex formation, which promotes liver cancer metastasis [41]. ZMYND8 acetylation at K1007 and K1034 is involved in mediating HIF-dependent breast cancer metastasis [42]. CBP-mediated DOT1L K358 acetylation promotes colorectal cancer metastasis by preventing DOT1L (an enzyme that catalyzes H3K79 methylation) from proteasomal degradation without affecting its enzyme activity [43]. SNAIL acetylation at K146 and K187 prevents its recognition by the E3 ubiquitin ligases FBXL14 and β-TRCP1 and thus reduces its ubiquitination and proteasomal degradation, whereas stabilized SNAIL promotes gastric cancer progression and metastasis [44]. On the contrary, the class I HDAC inhibitor MS-275 reduces sarcoma metastasis via enhancing rapid acetylation of YB-1 (Y-box RNA binding protein 1) at K81 and thus blocking the binding and translational activation of HIF1A, a YB-1 mRNA target [45]. HDAC6 (being also associated with non-histone protein modifications) suppresses hepatocellular carcinoma metastasis via enhancing α-tubulin acetylation at K40 [46].

### 3.3. Acetylation and Cancer Cell Metabolism

Acetylation dynamically interacts with cell metabolism as acetyl-CoA, the central metabolite, functions as the acetyl-donor for acetylation [47] (Figure 2). Acetylation is associated with cancer cell metabolism in many aspects including its role in metabolic homeostasis such as nutrient (e.g., glucose, lipid) and energy metabolism. 

Histone acetylation could be controlled by tuning the concentration of acetyl-CoA since the Michaelis constant (Km) of histone acetyltransferases (HATs) is within the physiological cellular concentration range of acetyl-CoA [48]. The interplay between histone acetylation and acetyl-CoA is influenced by many factors such as the amount and localization of enzymes, metabolites, and substrates that contribute to acetyl-CoA generation [48]. Metabolites such as glucose, lipids, and glutamine that can contribute to the global acetyl-CoA pool could increase histone acetylation, associating histone acetylation with nutrient metabolism. Specifically, citrate, as produced in mitochondria from glucose-derived carbon, travels into the nucleus where it is cleaved by ATP-citrate lyase (ACL, an enzyme converting glucose-derived citrate to acetyl-CoA) to produce acetyl-CoA for histone acetylation; and increased histone acetylation, in return, induces the expression of genes involved in glucose metabolism [49]. Lipid-derived carbon can lead to histone acetylation via lipid oxidation and lipid-derived acetyl-CoA [50,51]. Fatty acid synthesis and histone acetylation share the same acetyl-CoA pool as knocking down *ACC1* (encoding acetyl-CoA carboxylase, an enzyme playing critical roles in fatty acid synthesis) resulted in hyper-activated histone acetylation [52]. Furthermore, histone acetylation could impact the expression of genes required for reprogrammed ATP production, with ACL being the determining factor of the total amount of histone acetylation [49]. Perturbations of these acetyl-CoA-relevant metabolic programs could alter histone acetylation and consequently affect cancer progression. For instance, ACL nuclear translocation could increase histone acetylation and lead to up-regulated pyrimidine metabolism genes such as *DHODH* towards the enhanced endometrial tumor growth [53]; and suppressed H4 acetylation could lead to reduced lung cancer metastasis and lipid metabolism [54].

Nonhistone acetylation, if affecting the expression of TFs or enzymes that modulate cell metabolism, could reprogram cell metabolism. Aberrant acetylation could affect cancer cell initiation/progression via regulating the expression of genes, as well as the structure, stability, and localization of proteins relevant to these processes. For instance, O-linked N-acetylglucosamine transferase (OGT) could potentiate the expression of nuclear factor-κB (NFκB), a major glucose-responsive TF, in response to tumor necrosis factor stimulation by enhancing RelA acetylation at K310 [55]. Malic enzyme 1 (ME1) acetylation at K337 contributes to its dimerization and activation, leading to the enhanced nicotinamide adenine dinucleotide phosphate (NADPH) production, lipid metabolism, and colorectal tumorigenesis [56]. GNPAT acetylation at K128 represses its ubiquitination and protein degradation, and the stabilized GNPAT promotes lipid metabolism and hepatocarcinogenesis via inhibiting FASN degradation [57]. MnSOD acetylation at K68 destabilizes its superoxide-scavenging homotetramer to form a peroxidase-directed monomer, resulting in the chemotherapy and endocrine therapy resistance of estrogen receptor-positive (ER+) breast cancer cells as a result of mitochondrial metabolism reprogramming [58]. STAT3 acetylation at K685 triggers its mitochondria translocation to regulate the expression of energy metabolism-related genes [59].

### 3.4. Acetylation and Immunity 

Immunity, as comprised primarily of adaptive and innate immune responses, corresponds to the cancer hallmarks of ‘avoiding immune destruction’ and ‘tumor-promoting inflammation’, respectively.

As histone acetylation is sustained by acetyl-CoA [47] and metabolic regulation plays central roles in immune responses [60,61], histone acetylation has been implicated in the regulation of both adaptive and innate immunity. Regarding the adaptive immune response, fish oil influences the histone acetylation of neonatal T-cell genes implicated in adaptive immunity in a number of ways, such as (i) increasing H3 acetylation at *FOXP3*, *IL10RA*, *IL7R*, and H4 acetylation at *CD14* [62]; (ii) inducing LDHA, an enzyme converting pyruvate and NADH to lactate and NAD^+^, (iii) promoting INF_ϒ_ expression in activated T cells via elevating H3K9 acetylation, a histone mark of active transcription [63]; (iv) ligating glucocorticoid-induced TNFR-related protein (GITR), a costimulatory molecule of effect T cells and regulatory T cells (Tregs), on activated CD4^+^ T cells, which subverts the induction of FOXP3^+^ Tregs and directs these CD4^+^ T cells to Th9 cells towards boosted Th9 adaptive immune responses via *FOXP3* deacetylation at H3K9 and H3K27 [64]. Loss of H3K27 acetylation in the enhancer region of *CREBBP* leads to aberrant transcriptional silencing of genes that regulate B cell signaling and adaptive immune response including class II major histocompatibility complex (MHC) and consequently contribute to lymphomagenesis [65]. Regarding innate immune response involving histone acetylation, lipopolysaccharide (LPS) signaling directly promotes the incorporation of acetyl-CoA into histones, leading to enhanced H4K27 acetylation and induction of genes associated with inflammatory responses such as *IL-6*, *IL-12p40*, and *IL-12p70* [66]; inhibiting endothelial NOTCH1 signaling reduces cytokine-mediated H3K27 acetylation in a subset of NFκB-directed inflammatory enhancers that weaken endothelial up-regulation of adhesion molecules and recruit less leukocytes to the inflammatory sites [67].

Nonhistone acetylation, if residing in genes regulating core elements of the adaptive or innate immune response, could lead to aberrant immune and inflammatory responses. As examples of the adaptive immune response, pyruvate kinase M2 (PKM2) acetylation at K433 is critical for its de-tetramerization and nuclear translocation, where de-tetramerization helps activate dendritic cells as a result of its decreased enzymatic activity and nuclear localization makes it possible for PKM2 to form a complex with c-Rel towards the enhanced *Il12p35* expression and Th1 cell differentiation [68]; STAT3 acetylated at K87 binds BRD2 and is recruited to active enhancers occupied with TFs IRF4 and BATF, potentiating the genetic program required for Th17 cell differentiation and development [69]. Regarding examples on innate immune response, MKP1 acetylation at K57 reduces innate immune signaling and inflammation via blocking the MAPK pathway that plays pivotal roles in toll-like receptor signaling [70]; acetylation of α-tubulin at K40 is involved in augmentation of the NLRP3 inflammasome that enhances the innate immunity of the host [71]. Moreover, lysine acetylation of NKG2D ligand Rae-1 at K80 and K87 sensitizes tumor cells to NKG2D immune surveillance via NKG2D stabilization that involves both adaptive and innate immune responses [72].

### 3.5. Acetylation and Genome Instability 

Genome instability, as a common hallmark of cancers, is typically accompanied by aberrant acetylation signaling [73]. 

The DDR machinery plays fundamental roles in maintaining genome integrity. As histone acetylation modulates the structure of chromatin, controlling the organization and accessibility of the genome where many DDR activities occur, it orchestrates DDR activities as exemplified by the numerous histone acetylations such as H1K85 [74] and acetyl-lysine bromodomain proteins responsive to DNA damage [75,76,77,78]. In addition, HATs and HDACs are quite commonly involved in DDR and localized to the damaged loci [79]. For instance, KAT5 promotes homologous recombination (HR) in DNA double-strand break (DSB) repair by acetylating H4K16 [80] and H2AK15 [81] that block the recruitment of 53BP1 to the damaged chromatin; HDAC1/2 play critical roles in the DSB repair by deacetylating H3K56ac and H4K16ac [82]. Modulation of acetylation on non-histone proteins with critical roles in DDR also occurs frequently. For example, H3K56 and the DDR factor CtIP could both be deacetylated by SIRT6 during the DNA repair, with the first factor responsible for rapid SNF2H (the remodeling factor) recruitment to maintain genome stability [83] and the latter factor responsible for CtIP activation for DNA damage repair [84]; acetylation of PCNA at K20 promotes genome stability through stimulating HR [85].

Mutations gained from DNA transcription impose additional challenges to the genome stability. It has been proposed that cyclic histone acetylation and deacetylation of chromatin is a prerequisite for replication fidelity and crucial to prevent diverse spontaneous mutations [86]. For instance, H3K56 acetylation is abundant in the chromatin during the S phase and vanishes during the G2/M stage, where H3K56 acetylation is required for suppressing the spontaneous gross chromosomal rearrangement and, importantly, inhibiting spontaneous insertions/deletions and mutations together with mismatch repair and the proofreading activities of DNA replicative polymerases [86]. In addition, the functionalities of certain non-histone mismatch repair (MMR) proteins are governed by acetylation. For example, MutSα is composed of Msh2 and Msh6 and is a component of the complex (MutSα and MutSβ) responsible for DNA mismatch recognition; Msh2 acetylation at K845 and K847 stabilizes Msh6 [87], which in turn prevents Msh2 from ubiquitin-mediated protein degradation [88].

## 4. Acetylation-Mediated Crosstalk between Cancer Hallmarks and Epigenomic Events 

**Figure 3:**
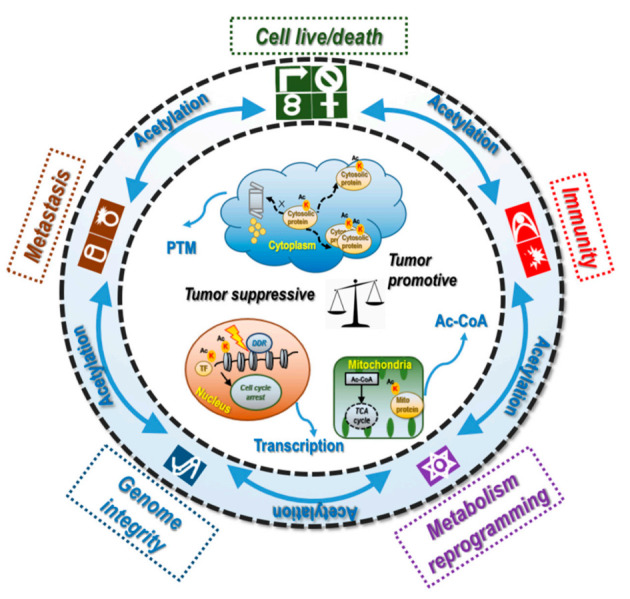
Crosstalk of cancer hallmarks as mediated via lysine acetylation. Cancer hallmarks develop crosstalk among themselves given the existence of some intrinsic connections such as the involvement of DDR in both cancer cell life/death control and genome integrity. Lysine acetylation plays critical roles in these dialogues as it can modulate the levels and functionalities of tumor suppressors and oncogenes involved in this cancer hallmark signaling at the transcriptional (nucleus), PTM (cytoplasm), and metabolic (mitochondria) levels.

Cancer hallmarks can crosstalk via lysine acetylation (Figure 3). For example, BRD4 functions in maintaining the growth and migration of gastric cancer cells through recognizing acetylated K146 and K187 on SNAIL to prevent SNAIL ubiquitination and degradation [44]. Cytochrome C acetylation at K53 in prostate cancer cells leads to enhanced Warburg effect and apoptosis evasion, enabling malignant cells with two cancer hallmarks [89,90]. Cyclic GMP-AMP synthase (cGAS) acetylation at K384 could trigger both downstream apoptosis and immune signaling [91]. The K20 acetylation of PCNA, a key factor in DNA replication and cell cycle regulation [92], is important for cell survival and promotes genome stability [85]. miRNA-15a-5p suppresses lung cancer cell migration/invasion and fatty acid synthesis by decreasing H4 acetylation [54]. The intrinsic connections between metabolic programming and innate/adaptive immune responses through acetyl-CoA make histone acetylation an excellent linker, as exemplified by the promotional role of aerobic glycolysis on Th1 cell differentiation through H3K9 acetylation [63]. WRN plays specific roles in DNA metabolism and genome stability, which is regulated by many PTM programs including acetylation [93]. Exosome-dependent immune surveillance at the metastatic niche requires p53 acetylation at K372 and K373 [94], and the activated miR193a/S100A6 axis suppresses the migration and proliferation of lung cancer cells via promoting p53 acetylation at K373 [95], where p53 is a well-known gatekeeper of genome integrity [96]. In addition, *ZEB1* ablation in stromal CAFs increases p53 acetylation at K382, which leads to its recruitment to *FGF2*, *FGF7*, *VEGF*, and *IL6* promoters and reduced production and secretion of these factors to the surrounding stroma. These effects lead to impaired extracellular matrix deposition and reduced recruitment of cancer-associated immune cells that suppress mammary epithelial cancer development [97].

**Figure 4:**
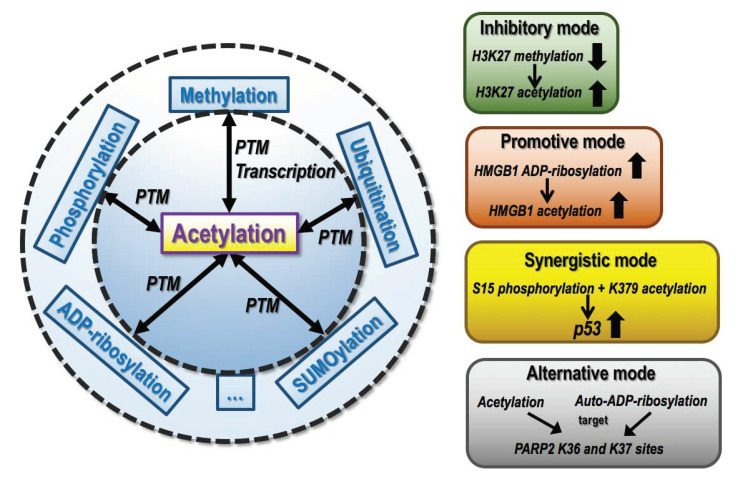
Crosstalk of lysine acetylation with other epigenetic events. Lysine acetylation develops crosstalk with other epigenetic events including, e.g., methylation (occurring at both transcriptional and PTM levels), and PTM events such as phosphorylation, ubiquitination, SUMOylation, and ADP-ribosylation to collectively regulate the levels and functionalities of genes involved in cancer hallmarks. There exist at least four modes among these crosstalks including the ‘inhibitory mode’, ‘promotional mode’, ‘synergistic mode’, ‘alternative mode’. An example of the ‘inhibitory mode’ is that reduced H3K27 methylation could lead to enhanced H3K27 acetylation. As an example of the ‘promotional mode’, ADP-ribosylation of HMGB1 could elevate its acetylation. An example of the ‘synergistic mode’ is that both S15 phosphorylation and K379 acetylation of p53 could lead to its enhanced expression. As an example of the ‘alternative mode’, the K36 and K37 of PARP2 are target sites of both acetylation and auto-ADP-ribosylation.

Lots of lysine residues are modifiable by multiple epigenetic programs including, e.g., acetylation, phosphorylation, methylation, ubiquitination, ADP-ribosylation, and SUMOylation, enabling the crosstalk of acetylation with other epigenomic events (Figure 4). For example, p53 is subjected to phosphorylation, ubiquitination, and SUMOylation in addition to acetylation, which collectively regulate its stability and transcriptional activity [98]; lovastatin, a potential candidate for breast cancer intervention, activates p53 via inducing both S15 phosphorylation and K379 acetylation [99]. Global loss of H3K27 methylation leads to abnormal accumulation of H3K27 acetylation at all sites where H3K27 methylation is lost; H3K27 hyper-acetylation induces the loss of PRC2 activity that is capable of triggering mono-, dual-, and triple- methylation of H3K27 [100]. H4K16 acetylation inhibits ADP-ribosylation that is mediated by PARP1 [101] and K36 and K37 sites of PARP2 are also targets of both acetylation and auto-ADP-ribosylation [102]. Additionally, PARP1 shares common lysine residues for acetylation, ubiquitination, and SUMOylation in Alzheimer’s and Parkinson’s diseases [103]. ADP-ribosylation is a PTM event that covalently attaches an ADP-ribose unit on lysine, arginine, serine, aspartate, and glutamate residues of target proteins using NAD^+^ as a substrate and is catalyzed by PARPs [104]; acetylation blocks ADP-ribosylation induced by DNA damage [105], and ADP-ribosylation of HMGB1 facilitates its acetylation in leukemia cells, which promotes chemotherapy-triggered cell autophagy [106]. A recent large-scale proteomic analysis identified 236 lysine residues from 141 proteins that are modified by both acetylation and ubiquitination using 13 representative human cancer cell lines from six different tissue types [107]. SUMOylation is a PTM event where a small ubiquitin-like modifier (SUMO) is covalently added to lysine residues; and inhibition of H3K23 deacetylation regulates breast cancer cell adhesion via triggering TRIM24 SUMOylation at K723 and K741 [108]. 

## 5. Combining HDAC Inhibitors with Cold Atmospheric Plasma Provides Novel Onco-Therapeutic Opportunities

HDAC isoenzymes were reported to be high in malignant cells [109]. HDAC inhibitors can reactivate the expression of tumor suppressors and have emerged as one type of well-characterized epigenome-targeting agents that are capable of suppressing both histone acetylation and non-histone acetylation to resolve tumors [110], halt cancer metastasis [111], reprogram cancer cell metabolism [112,113], modulate the immune [114], chemo [115] and radio [116] sensitivity of cancer cells. Several HDAC inhibitors have been developed and approved by the Food and Drug Administration (FDA) for cancer treatment that represent distinct HDAC specificity. For instance, vorinostat (Zolinza; Merck) [117], panobinostat (arydak; Novartis) [118], belinostat (Beleodap; Spectrum Pharmaceuticals) [119], and romidepsin (Istodax; Celgene) [120] have been approved for treating refractory multiple myeloma and cutaneous/peripheral T-cell lymphoma, where the first three drugs target HDAC1/2/3/6 and the last one inhibits HDAC1/2/3 [121].

Like other targeted therapies, cancer cells may also develop resistance to HDAC inhibitors due to, e.g., the use of alternative PTM programs shutting down gene transcription such as DNA methylation [122], the adoption of HSP90-induced unfolded protein response pathway, elevated stress-responsive TF such as NFκB (also named p65), enhanced level of anti-apoptotic proteins such as Bcl-2, and upregulated cellular anti-oxidant signaling [123]. These render it possible to combine HDAC inhibitors with other therapeutic strategies. By reviewing literatures on combinatorial strategies involving the four FDA-approved HDAC inhibitors vorinostat, panobinostat, belinostat, and romidepsin in the past 5 years, we found that inhibitors of kinases and their downstream effectors such as gefitinib, inducers of apoptosis and cell cycle arrest such as riluzole, and autophagy inhibitors such as quinacrine are typically combined with HDAC inhibitors for improved cell death; anti-angiogenetic agents such as bevacizumab and drugs targeting cancer stemness such as minocycline are commonly used together with HDAC inhibitors to halt metastasis; inhibitors of key enzymes involved in cell metabolisms such as simvastatin is primarily combined with HDAC inhibitors to target cancer cell metabolism; anti-inflammation agents such as dexamethasone, and drugs designed for immune-targeting such as rituximab are commonly used together with HDAC inhibitors towards reduced inflammation and enhanced immunotherapeutic efficacy; and alkylating agents such as cisplatin, topoisomerase inhibitors such as cytarabine, DNA synthesis inhibitors such as idarubicin, p53 activators such as CBL0137 are typically used together with HDAC inhibitors to toggle the genome integrity of cancer cells; inhibitors of HSP90 and proteosome such as 17AAG and ixazomib, DNA or histone methylation such as 5-azacytidine and 3-deazaneplanocin A can be jointly used with HDAC inhibitors to target any relevant cancer hallmark (Supplementary Table S1).

Cold atmospheric plasma (CAP), a cocktail of reactive oxygen and nitrogen species generated by ionization of gas via an electromagnetic field, has been proposed as an emerging therapeutic approach [124,125] with numerous studies demonstrating its selectivity against diverse cancer cells [126]. As CAP could selectively trigger apoptosis in many malignant cells [127,128,129], significantly reduce the amount of active NFκB that halts the migration of MDA-MB-231 triple-negative breast cancer (TNBCs) cells [126] and selectively eliminate more aggressive breast cancer cells showing mesenchymal attributes [129], target cancer cell metabolism [130], eliminate immunosuppressive pancreatic stellate cells and induce immunogenic cell death [131], and eradicate cancer stem cells that typically feature high anti-oxidative ability [132], the ability of CAP in targeting various cancer hallmarks is evident [125]. CAP has been considered as a relatively mild promising onco-therapeutic approach with minimal adverse effects on healthy cells [133]. Moreover, CAP is known to cause controlled oxidative stress [45,132] that can alter many cell death programs such as cell cycle progression and apoptosis [134]. To aid in the understanding of CAP as a novel therapeutic, recent progress on its other applications is summarized in Supplementary Table S2.

Importantly, it was demonstrated that CAP could be used to modify the human epigenome, with most reports focusing on DNA methylation, histone methylation, and histone acetylation [135]. Through pyrosequencing and whole-genome methylation microarray analysis, Park et al. reported the effect of CAP in triggering hypomethylation of a specific CpG site of *Alu* from 23.4 to 20.3% in MDAMB231 TNBC cells [136]. Hou et al. revealed the importance of exposure time in determining the epigenetic effect of CAP on cell signaling cascades through studying A549 small lung cancer cells; it was suggested that a 3 min or longer CAP exposure can possibly affect DNA methylation via altering methyltransferase activity [137]. The levels of many non-coding RNAs were reported to be affected by CAP such as miRNA-19a-3p [138] and ZNRD1-1AS1 lncRNA [139]; moreover, the effects of these non-coding RNAs were attributed to CAP-induced DNA methylation alteration in cancer cells as well. Lee et al. investigated the influence of CAP on histone methylation levels using the H3K4me3 (which is associated with activated gene expression) genome-wide ChIP-sequencing approach and showed that H3K4me3 levels were, in general, lower in CAP treated MCF7 cells as compared with the control [140]. Though relatively little has been reported on the effect of CAP on histone acetylation, CAP-treated human mesoderm-derived stem cells were characterized with the increased HDAC1 activity and decreased acetylated histone-3 levels [141]. On the other hand, synergistic killing effects of A549 non-small cell lung cancer cells were achieved by combining HDAC inhibitors and CAP [142]. These seemingly contradictory effects of CAP on histone acetylation in these two studies can be explained by the significant differences of cell signaling networks at the healthy and malignant states that may potentially justify the use of HDAC inhibitors with CAP for selective targeting of cancer cells. 

These demonstrated features of CAP not only make it a potential onco-therapeutic strategy but also an excellent remedy to prevent cancer cells from becoming resistant to HDAC inhibitors, rendering the joint use of HDAC inhibitors and CAP a novel and promising combinatorial strategy for cancer treatment. These possibilities require rigorous experimental investigations, among which whether CAP could function as an HSP90 inhibitor or HDAC inhibitor deserves the most attention.

## 6. Conclusions

Lysine acetylation plays multiple roles in many important cellular processes including gene expression, and the structure, activity, stability, and localization of proteins, and thus is involved in cancer initiation and progression. Its central position in linking the crosstalk among cancer hallmarks and PTM events further manifests its importance in carcinogenesis and underpins its promise in the design of novel onco-therapeutic strategies. CAP, being an emerging and selective (i.e., killing cancer but not healthy cells) onco-therapeutic modality with demonstrated efficacy against cancer hallmarks via redox modulation, represents a promising approach for combination with HDAC inhibitors for cancer treatment, with the potential for improved therapeutic efficacy and reduced adverse effects.

## Supplementary Materials

The following supporting information can be downloaded at: https://www.mdpi.com/article/10.3390/cancers14020346/s1, Table S1. Combinatorial onco-therapeutic modalities involving FDA-approved HDAC inhibitors belinostat, panobinostat, romidepsin, and vorinostat with a focus on the past 5 years (2017–2021); Table S2. Recent progress on other medical applications of CAP beyond its use as an onco-therapy.
