# Supplementary material for: Lysine Acetylation, Cancer Hallmarks and Emerging Onco-Therapeutic Opportunities
Source: Cancers (Basel). 2022 Jan 11;14(2):346. doi: 10.3390/cancers14020346 (PMC8773583; doi:10.3390/cancers14020346)
Supplement: Supplementary file 1 [file cancers-14-00346-s001.zip › cancers-1304850-supplementary.pdf]

*Review*

# Lysine Acetylation, Cancer Hallmarks and Emerging Onco-Therapeutic Opportunities

**Meilan Hu, Fu-le He, Erik W. Thompson, Kostya (Ken) Ostrikov and Xiaofeng Dai**

**Supplementary Table S1.** Combinatorial onco-therapeutic modalities involving FDA-approved HDAC inhibitors belinostat, panobinostat, romidepsin, vorinostat with a focus on the past 5 years (2017-2021).

| Drug composition. | Disease | Cancer hallmark | Mechanism | Clinical stage | Reference |
|---|---|---|---|---|---|
| Belinostat + Cisplatin + Etoposide | Small cell lung cancer | Genome integrity | HDAC(i) + alkylating agent + topoisomerase(i) | Phase I trial | [1] |
| Belinostat + Cyclophosphamide + Doxorubicin + Prednisone + Vincristine | Peripheral T-cell lymphoma | Genome integrity | HDAC(i) + alkylating agent + DNA synthesis(i) | Cohort study | [2] |
| Belinostat + Ixazomib | Hodgkin and T-cell lymphoma | Cancer cell live/death, Immune | HDAC(i) + proteasome(i) | Pre-clinical | [3] |
| Belinostat + Platinum | Non-small cell lung cancer | Genome integrity | HDAC(i) + alkylating agent | Pre-clinical | [4] |
| Belinostat + Pralatrexate | T-cell lymphoma, B-cell lymphoma | Metabolism | HDAC(i) + dihydrofolate reductase(i) | Pre-clinical | [5] |
| Belinostat + Radiotherapy | Rhabdomyosarcoma | Metastasis | HDAC(i) + radiation | Pre-clinical | [6] |
| Belinostat + Zevalin | Relapsed lymphoma | Immune | HDAC(i) + anti-CD20 antibody | Phase II trial | [7] |
| Belinostat + 13-cis-retinoic acid | Advanced solid cancer | Immune | HDAC(i) + anti-inflammation agent | Phase I trial | [8] |
| Belinostat + 17AAG | Triple negative breast cancer | Cancer cell live/death, Metastasis | HDAC(i) + HSP90(i) | Pre-clinical | [9] |
| Panobinostat + APR-246 + 3-deazaneplanocin A | Glioblastoma | Genome integrity | HDAC(i) + p53(a) + histone methylation(i) | Pre-clinical | [10] |
| Panobinostat + Azacitidine | Chronic myelomonocytic leukemia, acute myelocytic leukemia | Cancer cell live/death | HDAC(i) + DNA methylation(i) | Phase Ib/IIb trial | [11] |
| Panobinostat + BEZ235 | Glioblastoma | Immune | HDAC(i) + mTOR(i) | Pre-clinical | [12] |
| Panobinostat + BKM120 | Pre-B acute lymphoblastma | Cancer cell live/death | HDAC(i) + PI3K(i) | Pre-clinical | [13] |
| Panobinostat + Bicalutamide | Prostate cancer with castration resistance | Cancer cell live/death | HDAC(i) + AR(i) | Pre-clinical | [14] |
| Panobinostat + Bortezomib | Breast cancer, glioma | Cancer cell live/death, Immune, Metabolism | HDAC(i) + proteasome(i) | Pre-clinical | [15] |
| Panobinostat + Bortezomib + Dexamethasone | Relapsed myeloma | Immune, Metastasis | HDAC(i) + proteasome(i) + anti-inflammation agent | Phase II trial | [16] |
| Panobinostat + Bortezomib + Dexamethasone + Lenalidomide | Myeloma | Immune, Metastasis | HDAC(i) + proteasome(i) + anti-inflammation agent + ligand of ubiquitin E3 ligase | Phase I trial | [17] |
| Panobinostat + Carboplatin | Non-small cell lung cancer | Genome integrity | HDAC(i) + DNA synthesis(i) | Pre-clinical | [18] |
| Panobinostat + Carboplatin + Etoposide + Ifosfamide | Relapsed Hodgkin lymphoma | Genome integrity | HDAC(i) + DNA synthesis(i) + topoisomerase(i) + alkylating agent | Phase I trial | [19] |

| | | | | | |
|---|---|---|---|---|---|
| Panobinostat + Carfilzomib | Relapsed myeloma | Metastasis | HDAC(i) + proteasome(i) | Phase I/Ib/II trial | [20] |
| Panobinostat + CBL0137 | Neuroblastoma | Genome integrity | HDAC(i) + p53(a) | Pre-clinical | [21] |
| Panobinostat + Chloroquine | Ovarian cancer | Cancer cell live/death, Genome integrity | HDAC(i) + autophagy(i) | Pre-clinical | [22] |
| Panobinostat + Cytarabine/Doxorubicin | Acute myeloid leukemia | Genome integrity | HDAC(i) + topoisomerase(i) | Phase I trial | [23] |
| Panobinostat + Cytarabine + Idarubicin | Acute myeloid leukemia | Genome integrity | HDAC(i) + topoisomerase(i) + DNA/RNA synthesis(i) | Cohort study | [24] |
| Panobinostat + DZNEP + Temozolomide | Glioblastoma | Cancer cell live/death | HDAC(i) + histone methylation(i) + Alkylating agent | Pre-clinical | [25] |
| Panobinostat + Everolimus | Myeloma, advanced clear-cell renal cell carcinoma | Immune | HDAC(i) + mTOR(i) | Pre-clinical, Phase I trial | [26] |
| Panobinostat + Gefitinib | Non-small cell lung cancer with KRAS mutation | Cancer cell live/death | HDAC(i) + EGFR(i) | Pre-clinical | [27] |
| Panobinostat + Ibrutinib | Diffuse large B cell lymphoma with MyD88(L265P) mutation | Cancer cell live/death | HDAC(i) + TRK(i) | Pre-clinical | [28] |
| Panobinostat + Lenalidomide | Myeloma, relapsed Hodgkin lymphoma | Cancer cell live/death | HDAC(i) + ligand of ubiquitin E3 ligase | Pre-clinical, Phase I/II trial | [29] |
| Panobinostat + Melphalan + Prednisone + Thalidomide | Relapsed myeloma | Genome integrity, Immune | HDAC(i) + alkylating agent + anti-inflammation agent + TNFa biosynthesis(i) | Phase II trial | [30] |
| Panobinostat + Topotecan/Etoposide | Cervical cancer | Genome integrity | HDAC(i) + topoisomerase(i) | Pre-clinical | [31] |
| Panobinostat + 5-azacytidine | Chronic myelomonocytic leukemia | Cancer cell live/death | HDAC(i) + DNA methylation(i) | Phase I trial | [32] |
| Romidepsin + Alisertib | Relapsed B-cell lymphoma, Relapsed T-cell lymphoma | Cancer cell live/death | HDAC(i) + Aurora A(i) | Phase I trial | [33] |
| Romidepsin + Bendamustine | Relapsed T-cell lymphoma | Genome integrity | HDAC(i) + alkylating agent | Case study | [34] |
| Romidepsin + Bortezomib | Chronic lymphocytic lymphoma, indolent B-cell lymphoma, peripheral T-cell lymphoma, cutaneous T-cell lymphoma | Metastasis | HDAC(i) + proteasome(i) | Phase I trial | [35] |
| Romidepsin + CC486 | Advanced solid cancer | Cancer cell live/death | HDAC(i) + DNA methylation(i) | Phase I trial | [36] |
| Romidepsin + Cisplatin + Gemcitabine | Triple negative breast cancer, Urothelial carcinoma | Genome integrity | HDAC(i) + alkylating agent + DNA synthesis(i) | Pre-clinical | [37] |
| Romidepsin + Cisplatin + Dexamethasone + Gemcitabine | Peripheral T-cell lymphoma, diffuse large B-cell lymphoma | Genome integrity, Immune | HDAC(i) + alkylating agent + DNA synthesis(i) + anti-inflammation agent | Phase I/II trial | [38] |
| Romidepsin + Doxorubicin | Relapsed T-cell lymphoma | Genome integrity | HDAC(i) + topoisomerase(i) | Phase I trial | [39] |
| Romidepsin + Tamoxifen | T-cell lymphoma | Cancer cell live/death | HDAC(i) + growth inhibitory agent | Pre-clinical | [40] |
| Romidepsin + 5-azacytidine | Peripheral T-cell lymphoma | Cancer cell live/death | HDAC(i) + DNA methylation(i) | Phase I/II trial | [41] |

| | | | | | |
|---|---|---|---|---|---|
| Vorinostat + AA98 | Ovarian cancer | Immune | HDAC(i) + anti-CD146 antibody | Pre-clinical | [42] |
| Vorinostat + Alisertib | Lymphoid cancer | Cancer cell live/death | HDAC(i) + Aurora A(i) | Phase I trial | [43] |
| Vorinostat + Anti-GD2 antiboty | Aggressive orthotopic neuroblastoma | Immune | HDAC(i) + immune checkpoint antibody | Pre-clinical | [44] |
| Vorinostat + Anti-PD1 antibody + Sorafenib | Pancreatic cancer | Immune | HDAC(i) + immune checkpoint antibody + Raf(i) | Phase I trial | [45] |
| Vorinostat + Anti-oxidant therapy | Myeloproliferative neoplasms | Cancer cell live/death | HDAC(i) + anti-oxidant therapy | Pre-clinical | [46] |
| Vorinostat + Azacitidine | Chronic myelomonocytic leukemia | Cancer cell live/death | HDAC(i) + DNA methylation(i) | Phase II trial | [47] |
| Vorinostat + AZD1775 | Head and neck squamous cell carcinomas with p53 mutation | Genome integrity | HDAC(i) + Wee1(i) | Pre-clinical | [48] |
| Vorinostat + Bevacizumab | Clear-cell renal cell carcinoma, relapsed glioma | Metastasis | HDAC(i) + VEGFR(i) | Phase I/II trial | [49] |
| Vorinostat + Bevacizumab + Temozolomide | Relapsed glioma | Metastasis | HDAC(i) + VEGFR(i) + alkylating agent | Phase I/II trial | [50] |
| Vorinostat + Bortezomib | Myeloma, diffuse large B cell lymphoma, mantle cell lymphoma | Metastasis | HDAC(i) + proteasome(i) | Pre-clinical, Phase II trial | [51] |
| Vorinostat + Bortezomib + Dexamethasone | Relapsed myeloma | Genome integrity, Immune | HDAC(i) + proteasome(i) + anti-inflammation agent | Phase II trial | [52] |
| Vorinostat + Bortezomib + Dexamethasone + Doxorubicin | Relapsed myeloma | Genome integrity, Immune, Metastasis | HDAC(i) + proteasome(i) + anti-inflammation agent + topoisomerase(i) | Phase I/II trial | [53] |
| Vorinostat + Bortezomib + Dexamethasone + Lenalidomide | Myeloma | Immune | HDAC(i) + proteasome(i) + anti-inflammation agent + ligand of ubiquitin E3 ligase | Phase I trial | [54] |
| Vorinostat + Bortezomib + Doxorubicin | Relapsed myeloma | Genome integrity, Metastasis | HDAC(i) + proteasome(i) + topoisomerase(i) | Phase I trial | [55] |
| Vorinostat + Bortezomib + Sorafenib | Acute myeloid leukemia | Cancer cell live/death | HDAC(i) + proteasome(i) + RAF(i) | Phase I/II trial | [56] |
| Vorinostat + Brigatinib | Lung cancer with EGFR(C797S) mutation | Cancer cell live/death | HDAC(i) + TRK(i) | Pre-clinical | [57] |
| Vorinostat + Cladribine + Rituximab | Relapsed B-cell non-Hodgkin lymphoma, mantle cell lymphoma | Immune | HDAC(i) + adenosine deaminase(i) + anti-CD20 antibody | Phase I/II trial | [58] |
| Vorinostat + Cisplatin/5-Fluorouracil | Oral squamous cell carcinoma | Genome integrity | HDAC(i) + alkylating agent | Pre-clinical | [59] |
| Vorinostat + Conatumumab | Relapsed Hodgkin lymphoma | Cancer cell live/death | HDAC(i) + anti-DR5 antibody | Phase Ib trial | [60] |
| Vorinostat + Dexamethasone + Lenalidomide | Myeloma | Immune | HDAC(i) + anti-inflammation agent + ligand of ubiquitin E3 ligase | Phase IIb trial | [61] |
| Vorinostat + Disulfiram + PARP inhibitors | Prostate cancer | Genome integrity | HDAC(i) + DNA demethylation agent + PARP(i) | Pre-clinical | [62] |
| Vorinostat + Etoposide | Cervical cancer | Genome integrity | HDAC(i) + topoisomerase(i) | Pre-clinical | [63] |
| Vorinostat + Evodiamine | Hepatocellular cancer | Cancer cell live/death | HDAC(i) + TRPV1(a) | Pre-clinical | [64] |
| Vorinostat + Fenretinide | T-cell lymphoma | Cancer cell live/death | HDAC(i) + FAK(i) | Pre-clinical | [65] |

| | | | | | |
|---|---|---|---|---|---|
| Vorinostat + Gambogic acid | Neuroblastoma | Metastasis | HDAC(i) + anti-angiogenetic agent | Pre-clinical | [66] |
| Vorinostat + Gefitinib | Non-small cell lung cancer with EGFR mutation, lung cancer with EGFR mutation and BIM deletion | Cancer cell live/death | HDAC(i) + EGFR(i) | Pre-clinical, phase I trial | [67, 68] |
| Vorinostat + CM03 | Pancreatic cancer | Genome integrity | HDAC(i) + G-quadruplex-binding small molecule | Pre-clinical | [69] |
| Vorinostat + Isotretinoin | Advanced renal cell carcinoma, relapsed neuroblastoma | Cancer cell live/death | HDAC(i) + RAR(a) | Phase I trial | [70, 71] |
| Vorinostat + Ixabepilone | Metastatic breast cancer | Metastasis | HDAC(i) + proteasome(i) | Phase Ib trial | [72] |
| Vorinostat + Ixazomib/Pazopanib | Metastatic sarcoma or colorectal cancer with p53 mutation | Metastasis | HDAC(i) + proteasome(i) | Phase I trial | [73] |
| Vorinostat + Melatonin | Glioblastoma | Immune | HDAC(i) + IL1/2 producer | Pre-clinical | [74] |
| Vorinostat + Minocycline | Glioma | Metastasis | HDAC(i) + ALDH(i) | Pre-clinical | [75] |
| Vorinostat + Mithramycin A | Cutaneous T-cell lymphoma | Genome integrity | HDAC(i) + DNA synthesis(i) | Pre-clinical | [76] |
| Vorinostat + Oxaliplatin | Hepatocellular cancer | Genome integrity | HDAC(i) + alkylating agent | Pre-clinical | [77] |
| Vorinostat + Paclitaxel | Breast cancer | Cancer cell live/death | HDAC(i) + cell devision(i) | Pre-clinical | [78] |
| Vorinostat + Palbociclib | Mantle cell lymphoma with therapy resistance | Cancer cell live/death | HDAC(i) + CDK4/6(i) | Pre-clinical | [79] |
| Vorinostat + Pembrolizumab | Metastatic non-small cell lung cancer, relapsed head and neck squamous cell carcinomas, salivary gland cancer | Immune | HDAC(i) + anti-PD-L1 antibody | Phase I/Ib/II trial | [80] |
| Vorinostat + Quinacrine | T-cell acute lymphoblastic leukemia | Cancer cell live/death | HDAC(i) + autophagy(i) | Pre-clinical | [81] |
| Vorinostat + Radiation + Temozolomide | Glioblastoma | Genome integrity, Metastasis | HDAC(i) + radiation + alkylating agent | Phase I/II trial | [82] |
| Vorinostat + Riluzole | Breast cancer | Cancer cell live/death | HDAC(i) + cell cycle arrest inducer | Pre-clinical | [83] |
| Vorinostat + Rituximab-CHOP | Advanced diffuse large B-cell lymphoma | Immune | HDAC(i) + anti-CD20 antibody | Phase I/II trial | [84] |
| Vorinostat + Ruxolitinib | Myeloproliferative neoplasms, breast cancer, prostate cancer, colon cancer, hematological cancer | Immune | HDAC(i) + JAK/STAT signaling(i) | Pre-clinical | [85] |
| Vorinostat + Simvastatin | Triple negative breast cancer | Metabolism | HDAC(i) + HMG CoA reductase(i) | Pre-clinical | [86] |
| Vorinostat + Sorafenib | Advanced hepatocellular cancer | Cancer cell live/death | HDAC(i) + Raf-1/B-Raf(i) | Phase I trial | [87] |

Annotations: '(i)' and '(a)' each represents inhibitor and activator, respectively. Abbreviations: AR: androgen receptor; ALDH: aldehyde dehydrogenase; EGFR: epithelial growth factor receptor; FAK: focal adhesion kinase; IFNr: interferon gamma; RAR: retinoic acid receptor; TRK: tyrosine kinase; TNFa: tumor necrosis factor alpha; TRPV1: transient receptor potential vanilloid-1.

**Supplementary Table S2.** Recent progress on other medical applications of CAP beyond its use as an onco-therapy. Examples are given to show other properties of CAP enabling its medical applications beyond anti-cancer efficacy and has been intensively investigated in the past 5 years. In particular, CAP could kill bacteria including antibiotic-resistant strains, mixed species biofilms, endospores, viruses and prions that enable its 'decontamination' feature. Importantly, CAP was recently found to boost virus multiplication towards enhanced cell-based vaccine production that opens a novel avenue for CAP medical application.

| Applications | Details | CAP features | References |
|---|---|---|---|
| Infectious disease | SARS-CoV-2 control | Decontamination | [88–90] |
| Dentistry | Dental caries removal | Decontamination | [91,92] |
| | Implant disinfection | Decontamination | [93,94] |
| | Root canal disinfection | Decontamination | [91,95] |
| | Tooth bleaching | Surface modification | [96,97] |
| | Dentin treatment for ethetics | Surface modification | [98–101] |
| Surgery | Hemostasis | Wound healing | [102] |
| Diabetic foot | Diabetic foot ulcer treatment | Wound healing | [103] |
| Vaccine | Boost vaccine production | Virus titer enhancement | [104] |

## References


[1] S. Balasubramaniam, C.E. Redon, C.J. Peer, C. Bryla, M.J. Lee, J.B. Trepel, Y. Tomita, A. Rajan, G. Giaccone, W.M. Bonner, W.D. Figg, T. Fojo, R.L. Piekarz, S.E. Bates, Phase I trial of belinostat with cisplatin and etoposide in advanced solid tumors, with a focus on neuroendocrine and small cell cancers of the lung, Anticancer Drugs 29(5) (2018) 457-465.

[2] P.B. Johnston, A.F. Cashen, P.G. Nikolinakos, A.W. Beaven, S.K. Barta, G. Bhat, S.J. Hasal, S. De Vos, Y. Oki, C. Deng, F.M. Foss, Belinostat in combination with standard cyclophosphamide, doxorubicin, vincristine and prednisone as first-line treatment for patients with newly diagnosed peripheral T-cell lymphoma, Exp Hematol Oncol 10(1) (2021) 15.

[3] F.C. Passero, Jr., D. Ravi, J.T. McDonald, A. Beheshti, K.A. David, A.M. Evens, Combinatorial ixazomib and belinostat therapy induces NFE2L2-dependent apoptosis in Hodgkin and T-cell lymphoma, Br J Haematol 188(2) (2020) 295-308.

[4] K.K. To, W.S. Tong, L.W. Fu, Reversal of platinum drug resistance by the histone deacetylase inhibitor belinostat, Lung Cancer 103 (2017) 58-65.

[5] G.J. Peters, F.P.A. van Gemert, I. Kathmann, G. Reddy, S. Cillessen, G. Jansen, Schedule-Dependent Synergy Between the Histone Deacetylase Inhibitor Belinostat and the Dihydrofolate Reductase Inhibitor Pralatrexate in T-and B-cell Lymphoma Cells in vitro, Front Cell Dev Biol 8 (2020) 577215.

[6] F. Marampon, V. Di Nisio, I. Pietrantoni, F. Petragnano, I. Fasciani, B.M. Scicchitano, C. Ciccarelli, G.L. Gravina, C. Festuccia, A. Del Fattore, M. Tombolini, F. De Felice, D. Musio, S. Cecconi, P. Tini, M. Maddalo, S. Codenotti, A. Fanzani, A. Polimeni, R. Maggio, V. Tombolini, Pro-differentiating and radiosensitizing effects of inhibiting HDACs by PXD-101 (Belinostat) in in vitro and in vivo models of human rhabdomyosarcoma cell lines, Cancer Lett 461 (2019) 90-101.

[7] S.D. Puvvada, J.M. Guillen-Rodriguez, X.I. Rivera, K. Heard, L. Inclan, M. Schmelz, J.H. Schatz, D.O. Persky, A Phase II Exploratory Study of PXD-101 (Belinostat) Followed by Zevalin in Patients with Relapsed Aggressive High-Risk Lymphoma, Oncology 93(6) (2017) 401-405.

[8] T. Luu, P. Frankel, J.H. Beumer, D. Lim, M. Cristea, L.J. Appleman, H.J. Lenz, D.R. Gandara, B.F. Kiesel, R.L. Piekarz, E.M. Newman, Phase I trial of belinostat in combination with 13-cis-retinoic acid in advanced solid tumor malignancies: a California Cancer Consortium NCI/CTEP sponsored trial, Cancer Chemother Pharmacol 84(6) (2019) 1201-1208.

[9] Y. Zuo, H. Xu, Z. Chen, F. Xiong, B. Zhang, K. Chen, H. Jiang, C. Luo, H. Zhang, 17AAG synergizes with Belinostat to exhibit a negative effect on the proliferation and invasion of MDAMB231 breast cancer cells, Oncol Rep 43(6) (2020) 1928-1944.

[10] J. De La Rosa, A. Urdiciain, M.V. Zelaya, I. Zazpe, B. Melendez, J.A. Rey, M.A. Idoate, J.S. Castresana, APR-246 combined with 3-deazaneplanocin A, panobinostat or temozolomide reduces clonogenicity and induces apoptosis in glioblastoma cells, Int J Oncol 58(3) (2021) 312-330.

[11] G. Garcia-Manero, M.A. Sekeres, M. Egyed, M. Breccia, C. Graux, J.D. Cavenagh, H. Salman, A. Illes, P. Fenaux, D.J. DeAngelo, R. Stauder, K. Yee, N. Zhu, J.H. Lee, D. Valcarcel, A. MacWhannell, Z. Borbenyi, L. Gazi, S. Acharyya, S. Ide, M. Marker, O.G. Ottmann, A phase 1b/2b multicenter study of oral panobinostat plus azacitidine in adults with MDS, CMML or AML with 30% blasts, Leukemia 31(12) (2017) 2799-2806.

[12] W. Meng, B. Wang, W. Mao, J. Wang, Y. Zhao, Q. Li, C. Zhang, J. Ma, Enhanced efficacy of histone deacetylase inhibitor panobinostat combined with dual PI3K/mTOR inhibitor BEZ235 against glioblastoma, Nagoya J Med Sci 81(1) (2019) 93-102.

[13] M. Mehrpouri, M. Momeny, D. Bashash, Synergistic effects of BKM120 and panobinostat on pre-B acute lymphoblastic cells: an emerging perspective for the simultaneous inhibition of PI3K and HDACs, J Recept Signal Transduct Res (2020) 1-9.
[14] A.C. Ferrari, J.J. Alumkal, M.N. Stein, M.E. Taplin, J. Babb, E.S. Barnett, A. Gomez-Pinillos, X. Liu, D. Moore, R. DiPaola, T.M. Beer, Epigenetic Therapy with Panobinostat Combined with Bicalutamide Rechallenge in Castration-Resistant Prostate Cancer, Clin Cancer Res 25(1) (2019) 52-63.
[15] E.P. Jane, D.R. Premkumar, S. Thambireddy, B. Golbourn, S. Agnihotri, K.C. Bertrand, S.C. Mack, M.I. Myers, A. Chattopadhyay, D.L. Taylor, M.E. Schurdak, A.M. Stern, I.F. Pollack, Targeting NAD(+) Biosynthesis Overcomes Panobinostat and Bortezomib-Induced Malignant Glioma Resistance, Mol Cancer Res 18(7) (2020) 1004-1017.
[16] J.P. Laubach, F. Schjesvold, M. Mariz, M.A. Dimopoulos, E. Lech-Maranda, I. Spicka, V.T.M. Hungria, T. Shelekhova, A. Abdo, L. Jacobasch, C. Polprasert, R. Hajek, A. Illes, T. Wrobel, A. Sureda, M. Beksac, I.Z. Goncalves, J. Blade, S.V. Rajkumar, A. Chari, S. Lonial, A. Spencer, P. Maison-Blanche, P. Moreau, J.F. San-Miguel, P.G. Richardson, Efficacy and safety of oral panobinostat plus subcutaneous bortezomib and oral dexamethasone in patients with relapsed or relapsed and refractory multiple myeloma (PANORAMA 3): an open-label, randomised, phase 2 study, Lancet Oncol 22(1) (2021) 142-154.
[17] E.E. Manasanch, J.J. Shah, H.C. Lee, D.M. Weber, S.K. Thomas, B. Amini, L. Feng, Z. Berkova, M. Hildebrandt, R.Z. Orlowski, Bortezomib, lenalidomide, and dexamethasone with panobinostat for front-line treatment of patients with multiple myeloma who are eligible for transplantation: a phase 1 trial, Lancet Haematol 5(12) (2018) e628-e640.
[18] L. Wang, N.L. Syn, V.V. Subhash, Y. Any, W.L. Thuya, E.S.H. Cheow, L. Kong, F. Yu, P.C. Peethala, A.L. Wong, H.J. Laljibhai, A. Chinnathambi, P.S. Ong, P.C. Ho, G. Sethi, W.P. Yong, B.C. Goh, Pan-HDAC inhibition by panobinostat mediates chemosensitization to carboplatin in non-small cell lung cancer via attenuation of EGFR signaling, Cancer Lett 417 (2018) 152-160.
[19] B. Hu, A. Younes, J.R. Westin, F. Turturro, L. Claret, L. Feng, N. Fowler, S. Neelapu, J. Romaguera, F.B. Hagemeister, M.A. Rodriguez, F. Samaniego, L.E. Fayad, A.R. Copeland, L.J. Nastoupil, Y. Nieto, M.A. Fanale, Y. Oki, Phase-I and randomized phase-II trial of panobinostat in combination with ICE (ifosfamide, carboplatin, etoposide) in relapsed or refractory classical Hodgkin lymphoma, Leuk Lymphoma 59(4) (2018) 863-870.
[20] E.E. Manasanch, J.J. Shah, H.C. Lee, D.M. Weber, S.K. Thomas, B. Amini, J. Olsem, B. Crumpton, A. Morphey, Z. Berkova, L. Feng, R.Z. Orlowski, Phase I/Ib study of carfilzomib and panobinostat with or without dexamethasone in patients with relapsed/refractory multiple myeloma, Haematologica 105(5) (2020) e242-e245.
[21] L. Xiao, K. Somers, J.E. Murray, R. Pandher, M. Karsa, E. Ronca, A. Bongers, R. Terry, A. Ehteda, L.D. Gamble, N. Issaeva, K.I. Leonova, A. O'Connor, C. Mayoh, P. Venkat, H. Quek, J. Brand, F.K. Kusuma, J.A. Pettitt, E. Mosmann, A. Kearns, G. Eden, S. Alfred, S. Allan, L. Zhai, A. Kamili, A.J. Gifford, D.R. Carter, M.J. Henderson, J.I. Fletcher, G. Marshall, R.W. Johnstone, A.J. Cesare, D.S. Ziegler, A.V. Gudkov, K.V. Gurova, M.D. Norris, M. Haber, Dual targeting of chromatin stability by the curaxin CBL0137 and histone deacetylase inhibitor panobinostat shows significant preclinical efficacy in neuroblastoma, Clin Cancer Res (2021).
[22] M. Ovejero-Sanchez, R. Gonzalez-Sarmiento, A.B. Herrero, Synergistic effect of Chloroquine and Panobinostat in ovarian cancer through induction of DNA damage and inhibition of DNA repair, Neoplasia 23(5) (2021) 515-528.
[23] M.J. Wieduwilt, N. Pawlowska, S. Thomas, R. Olin, A.C. Logan, L.E. Damon, T. Martin, M. Kang, P.H. Sayre, W. Boyer, K.M.L. Gaensler, K. Anderson, P.N. Munster, C. Andreadis, Histone Deacetylase Inhibition with Panobinostat Combined with Intensive Induction Chemotherapy in Older Patients with Acute Myeloid Leukemia: Phase I Study Results, Clin Cancer Res 25(16) (2019) 4917-4923.
[24] D.J. DeAngelo, A.R. Walker, R.F. Schlenk, J. Sierra, B.C. Medeiros, E.M. Ocio, C. Rollig, S.A. Strickland, F. Thol, S.Z. Valera, K. Dasgupta, N. Berkowitz, R.K. Stuart, Safety and efficacy of oral panobinostat plus chemotherapy in patients aged 65 years or younger with high-risk acute myeloid leukemia, Leuk Res 85 (2019) 106197.
[25] J. De La Rosa, A. Urdiciain, I. Zazpe, M.V. Zelaya, B. Melendez, J.A. Rey, M.A. Idoate, J.S. Castresana, The synergistic effect of DZNEP, panobinostat and temozolomide reduces clonogenicity and induces apoptosis in glioblastoma cells, Int J Oncol 56(1) (2020) 283-300.
[26] A. Wood, S. George, N. Adra, S. Chintala, N. Damayanti, R. Pili, Phase I study of the mTOR inhibitor everolimus in combination with the histone deacetylase inhibitor panobinostat in patients with advanced clear cell renal cell carcinoma, Invest New Drugs 38(4) (2020) 1108-1116.
[27] W.Y. Lee, P.C. Chen, W.S. Wu, H.C. Wu, C.H. Lan, Y.H. Huang, C.H. Cheng, K.C. Chen, C.W. Lin, Panobinostat sensitizes KRAS-mutant non-small-cell lung cancer to gefitinib by targeting TAZ, Int J Cancer 141(9) (2017) 1921-1931.

[28] P. Mondello, E.J. Brea, E. De Stanchina, E. Toska, A.Y. Chang, M. Fennell, V. Seshan, R. Garippa, D.A. Scheinberg, J. Baselga, H.G. Wendel, A. Younes, Panobinostat acts synergistically with ibrutinib in diffuse large B cell lymphoma cells with MyD88 L265P mutations, JCI Insight 3(22) (2018).

[29] S. Tang, D. Ma, B. Cheng, Q. Fang, X. Kuang, K. Yu, W. Wang, B. Hu, J. Wang, Crucial role of HO-1/IRF4-dependent apoptosis induced by panobinostat and lenalidomide in multiple myeloma, Exp Cell Res 363(2) (2018) 196-207.

[30] M. Offidani, L. Corvatta, A.M. Liberati, S. Pulini, S. Ballanti, S. Bringhen, Updated results of a phase 2 study of panobinostat combined with melphalan, thalidomide and prednisone (MPT) in relapsed/refractory multiple myeloma, Leuk Lymphoma 59(5) (2018) 1271-1273.

[31] L. Wasim, M. Chopra, Synergistic anticancer effect of panobinostat and topoisomerase inhibitors through ROS generation and intrinsic apoptotic pathway induction in cervical cancer cells, Cell Oncol (Dordr) 41(2) (2018) 201-212.

[32] Y. Kobayashi, W. Munakata, M. Ogura, T. Uchida, M. Taniwaki, T. Kobayashi, F. Shimada, M. Yonemura, F. Matsuoka, T. Tajima, K. Yakushijin, H. Minami, Phase I study of panobinostat and 5-azacitidine in Japanese patients with myelodysplastic syndrome or chronic myelomonocytic leukemia, Int J Hematol 107(1) (2018) 83-91.

[33] P. Strati, L.J. Nastoupil, R.E. Davis, L.E. Fayad, N. Fowler, F.B. Hagemeister, L. Kwak, Y. Oki, M. Wang, J. Westin, C.E. Ruben, E.T. Wesson, R. Piekarz, M.A. Fanale, H.J. Lee, A phase 1 trial of alisertib and romidepsin for relapsed/refractory aggressive B-cell and T-cell lymphomas, Haematologica 105(1) (2020) e26-e28.

[34] B. Nachmias, A. Shaulov, D. Lavie, N. Goldschmidt, A. Gural, R. Saban, E. Lebel, M.E. Gatt, Romidepsin-Bendamustine Combination for Relapsed/Refractory T Cell Lymphoma, Acta Haematol 141(4) (2019) 216-221.

[35] B. Holkova, V. Yazbeck, M. Kmieciak, P. Bose, S. Ma, A. Kimball, M.B. Tombes, E. Shrader, W. Wan, C. Weir-Wiggins, A. Singh, K.T. Hogan, S. Conine, H. Sankala, J.D. Roberts, T.C. Shea, S. Grant, A phase 1 study of bortezomib and romidepsin in patients with chronic lymphocytic leukemia/small lymphocytic lymphoma, indolent B-cell lymphoma, peripheral T-cell lymphoma, or cutaneous T-cell lymphoma, Leuk Lymphoma 58(6) (2017) 1349-1357.

[36] S.L. Gaillard, M. Zahurak, A. Sharma, J.N. Durham, K.A. Reiss, S. Sartorius-Mergenthaler, M. Downs, N.M. Anders, N. Ahuja, M.A. Rudek, N. Azad, A phase 1 trial of the oral DNA methyltransferase inhibitor CC-486 and the histone deacetylase inhibitor romidepsin in advanced solid tumors, Cancer 125(16) (2019) 2837-2845.

[37] P. Pattarawat, T. Hong, S. Wallace, Y. Hu, R. Donnell, T.H. Wang, C.L. Tsai, J. Wang, H.R. Wang, Compensatory combination of romidepsin with gemcitabine and cisplatin to effectively and safely control urothelial carcinoma, Br J Cancer 123(2) (2020) 226-239.

[38] T. Reiman, K.J. Savage, M. Crump, M.C. Cheung, D. MacDonald, R. Buckstein, S. Couban, E. Piliotis, K. Imrie, D. Spaner, S. Shivakumar, J. Kuruvilla, D. Villa, L.E. Shepherd, T. Skamene, C. Winch, B.E. Chen, A.E. Hay, A phase I study of romidepsin, gemcitabine, dexamethasone and cisplatin combination therapy in the treatment of peripheral T-cell and diffuse large B-cell lymphoma; the Canadian cancer trials group LY.15 studydagger, Leuk Lymphoma 60(4) (2019) 912-919.

[39] K. Vu, C.H. Wu, C.Y. Yang, A. Zhan, E. Cavallone, W. Berry, P. Heeter, L. Pincus, M.J. Wieduwilt, B.M. William, C. Andreadis, L.K. Kaplan, F. McCormick, P. Porcu, J.E. Brammer, W.Z. Ai, Romidepsin Plus Liposomal Doxorubicin Is Safe and Effective in Patients with Relapsed or Refractory T-Cell Lymphoma: Results of a Phase I Dose-Escalation Study, Clin Cancer Res 26(5) (2020) 1000-1008.

[40] C. Wanitpongpun, Y. Honma, T. Okada, R. Suzuki, U. Takeshi, J. Suzumiya, Tamoxifen enhances romidepsin-induced apoptosis in T-cell malignant cells via activation of FOXO1 signaling pathway, Leuk Lymphoma (2021) 1-15.

[41] L. Falchi, H. Ma, S. Klein, J.K. Lue, F. Montanari, E. Marchi, C. Deng, H.A. Kim, A. Rada, A.T. Jacob, C. Kinahan, M.M. Francescone, C.R. Soderquist, D.C. Park, G. Bhagat, R. Nandakumar, D. Menezes, L. Scotto, L. Sokol, A.R. Shustov, O.A. O'Connor, Combined oral 5-azacytidine and romidepsin are highly effective in patients with PTCL: a multicenter phase 2 study, Blood 137(16) (2021) 2161-2170.

[42] X. Ma, J. Wang, J. Liu, Q. Mo, X. Yan, D. Ma, H. Duan, Targeting CD146 in combination with vorinostat for the treatment of ovarian cancer cells, Oncol Lett 13(3) (2017) 1681-1687.

[43] T. Siddiqi, P. Frankel, J.H. Beumer, B.F. Kiesel, S. Christner, C. Ruel, J.Y. Song, R. Chen, K.R. Kelly, S. Ailawadhi, P. Kaesberg, L. Popplewell, S. Puverel, R. Piekarz, S.J. Forman, E.M. Newman, Phase 1 study of the Aurora kinase A inhibitor alisertib (MLN8237) combined with the histone deacetylase inhibitor vorinostat in lymphoid malignancies, Leuk Lymphoma 61(2) (2020) 309-317.

[44] R.J.E. van den Bijgaart, M. Kroesen, I.C. Brok, D. Reijnen, M. Wassink, L. Boon, P.M. Hoogerbrugge, G.J. Adema, Anti-GD2 antibody and Vorinostat immunocombination therapy is highly effective in an aggressive orthotopic neuroblastoma model, Oncoimmunology 9(1) (2020) 1817653.

[45] L. Booth, J.L. Roberts, A. Poklepovic, P. Dent, Prior exposure of pancreatic tumors to [sorafenib + vorinostat] enhances the efficacy of an anti-PD-1 antibody, Cancer Biol Ther 20(1) (2019) 109-121.

[46] B.A. Cardoso, T.L. Ramos, H. Belo, F. Vilas-Boas, C. Real, A.M. Almeida, Vorinostat synergizes with antioxidant therapy to target myeloproliferative neoplasms, Exp Hematol 72 (2019) 60-71 e11.

[47] M.A. Sekeres, M. Othus, A.F. List, O. Odenike, R.M. Stone, S.D. Gore, M.R. Litzow, R. Buckstein, M. Fang, D. Roulston, C.D. Bloomfield, A. Moseley, A. Nazha, Y. Zhang, M.R. Velasco, R. Gaur, E. Atallah, E.C. Attar, E.K. Cook, A.H. Cull, M.J. Rauh, F.R. Appelbaum, H.P. Erba, Randomized Phase II Study of Azacitidine Alone or in Combination With Lenalidomide or With Vorinostat in Higher-Risk Myelodysplastic Syndromes and Chronic Myelomonocytic Leukemia: North American Intergroup Study SWOG S1117, J Clin Oncol 35(24) (2017) 2745-2753.

[48] N. Tanaka, A.A. Patel, L. Tang, N.L. Silver, A. Lindemann, H. Takahashi, R. Jaksik, X. Rao, N.N. Kalu, T.C. Chen, J. Wang, M.J. Frederick, F. Johnson, F.O. Gleber-Netto, S. Fu, M. Kimmel, J. Wang, W.N. Hittelman, C.R. Pickering, J.N. Myers, A.A. Osman, Replication Stress Leading to Apoptosis within the S-phase Contributes to Synergism between Vorinostat and AZD1775 in HNSCC Harboring High-Risk TP53 Mutation, Clin Cancer Res 23(21) (2017) 6541-6554.

[49] A. Ghiaseddin, D. Reardon, W. Massey, A. Mannerino, E.S. Lipp, J.E. Herndon, 2nd, F. McSherry, A. Desjardins, D. Randazzo, H.S. Friedman, K.B. Peters, Phase II Study of Bevacizumab and Vorinostat for Patients with Recurrent World Health Organization Grade 4 Malignant Glioma, Oncologist 23(2) (2018) 157-e21.

[50] K.B. Peters, E.S. Lipp, E. Miller, J.E. Herndon, 2nd, F. McSherry, A. Desjardins, D.A. Reardon, H.S. Friedman, Phase I/II trial of vorinostat, bevacizumab, and daily temozolomide for recurrent malignant gliomas, J Neurooncol 137(2) (2018) 349-356.

[51] C. Nanavati, D.E. Mager, Sequential Exposure of Bortezomib and Vorinostat is Synergistic in Multiple Myeloma Cells, Pharm Res 34(3) (2017) 668-679.

[52] S. Brown, C. Pawlyn, A.L. Tillotson, D. Sherratt, L. Flanagan, E. Low, G.J. Morgan, C. Williams, M. Kaiser, F.E. Davies, M.W. Jenner, U.K.E.P.C.T.N. Myeloma, Bortezomib, Vorinostat, and Dexamethasone Combination Therapy in Relapsed Myeloma: Results of the Phase 2 MUK four Trial, Clin Lymphoma Myeloma Leuk 21(3) (2021) 154-161 e3.

[53] J.M. Waldschmidt, A. Keller, G. Ihorst, O. Grishina, S. Muller, D. Wider, A.V. Frey, K. King, R. Simon, A. May, P. Tassone, J. Duyster, M. Jung, N. Raje, R. Wasch, M. Engelhardt, Safety and efficacy of vorinostat, bortezomib, doxorubicin and dexamethasone in a phase I/II study for relapsed or refractory multiple myeloma (VERUMM study: vorinostat in elderly, relapsed and unfit multiple myeloma), Haematologica 103(10) (2018) e473-e479.

[54] J.L. Kaufman, R. Mina, J.J. Shah, J.P. Laubach, A.K. Nooka, C. Lewis, C. Gleason, C. Sharp, R.D. Harvey, L.T. Heffner, P. Richardson, S. Lonial, R.Z. Orlowski, Phase 1 Trial Evaluating Vorinostat Plus Bortezomib, Lenalidomide, and Dexamethasone in Patients With Newly Diagnosed Multiple Myeloma, Clin Lymphoma Myeloma Leuk 20(12) (2020) 797-803.

[55] P.M. Voorhees, C. Gasparetto, D.T. Moore, D. Winans, R.Z. Orlowski, D.D. Hurd, Final Results of a Phase 1 Study of Vorinostat, Pegylated Liposomal Doxorubicin, and Bortezomib in Relapsed or Refractory Multiple Myeloma, Clin Lymphoma Myeloma Leuk 17(7) (2017) 424-432.

[56] H. Sayar, L.D. Cripe, A.N. Saliba, M. Abu Zaid, H. Konig, H.S. Boswell, Combination of sorafenib, vorinostat and bortezomib for the treatment of poor-risk AML: report of two consecutive clinical trials, Leuk Res 77 (2019) 30-33.

[57] C.Y. Lin, K.Y. Huang, Y.C. Lin, S.C. Yang, W.C. Chung, Y.L. Chang, J.Y. Shih, C.C. Ho, C.A. Lin, C.C. Shih, Y.H. Chang, S.H. Kao, P.C. Yang, Vorinostat combined with brigatinib overcomes acquired resistance in EGFR-C797S-mutated lung cancer, Cancer Lett 508 (2021) 76-91.

[58] S.E. Spurgeon, K. Sharma, D.F. Claxton, C. Ehmann, J. Pu, S. Shimko, A. Stewart, N. Subbiah, G. Palmbach, F. LeBlanc, E. Latour, Y. Chen, M. Mori, Z. Hasanali, E.M. Epner, Phase 1-2 study of vorinostat (SAHA), cladribine and rituximab (SCR) in relapsed B-cell non-Hodgkin lymphoma and previously untreated mantle cell lymphoma, Br J Haematol 186(6) (2019) 845-854.

[59] G. Piro, M.S. Roca, F. Bruzzese, C. Carbone, F. Iannelli, A. Leone, M.G. Volpe, A. Budillon, E. Di Gennaro, Vorinostat Potentiates 5-Fluorouracil/Cisplatin Combination by Inhibiting Chemotherapy-Induced EGFR Nuclear Translocation and Increasing Cisplatin Uptake, Mol Cancer Ther 18(8) (2019) 1405-1417.

[60] W.P.t. Skelton, E. Turba, L. Sokol, Durable Complete Response to AMG 655 (Conatumumab) and Vorinostat in a Patient With Relapsed Classical Hodgkin Lymphoma: Extraordinary Response from a Phase 1b Clinical Protocol, Clin Lymphoma Myeloma Leuk 20(12) (2020) e944-e946.

[61] L. Sanchez, D.H. Vesole, J.R. Richter, N. Biran, E. Bilotti, L. McBride, P. Anand, K. Ivanovski, D.S. Siegel, A phase IIb trial of vorinostat in combination with lenalidomide and dexamethasone in patients with multiple myeloma refractory to previous lenalidomide-containing regimens, Br J Haematol 176(3) (2017) 440-447.

[62] D. Majera, Z. Skrott, J. Bouchal, J. Bartkova, D. Simkova, M. Gachechiladze, J. Steigerova, D. Kurfurstova, J. Gursky, G. Korinkova, K. Cwiertka, Z. Hodny, M. Mistrik, J. Bartek, Targeting genotoxic and proteotoxic

stress-response pathways in human prostate cancer by clinically available PARP inhibitors, vorinostat and disulfiram, Prostate 79(4) (2019) 352-362.
[63] P. Kumar, L. Wasim, M. Chopra, A. Chhikara, Co-delivery of Vorinostat and Etoposide Via Disulfide Cross-Linked Biodegradable Polymeric Nanogels: Synthesis, Characterization, Biodegradation, and Anticancer Activity, AAPS PharmSciTech 19(2) (2018) 634-647.
[64] Y.L. Li, N.Y. Zhang, X. Hu, J.L. Chen, M.J. Rao, L.W. Wu, Q.Y. Li, B. Zhang, W. Yan, C. Zhang, Evodiamine induces apoptosis and promotes hepatocellular carcinoma cell death induced by vorinostat via downregulating HIF-1alpha under hypoxia, Biochem Biophys Res Commun 498(3) (2018) 481-486.
[65] M.R. Makena, T.H. Nguyen, B. Koneru, A. Hindle, W.H. Chen, D.U. Verlekar, M.H. Kang, C.P. Reynolds, Vorinostat and fenretinide synergize in preclinical models of T-cell lymphoid malignancies, Anticancer Drugs 32(1) (2021) 34-43.
[66] K. Bishayee, K. Habib, A. Sadra, S.O. Huh, Targeting the Difficult-to-Drug CD71 and MYCN with Gambogic Acid and Vorinostat in a Class of Neuroblastomas, Cell Physiol Biochem 53(1) (2019) 258-280.
[67] S.E. Park, D.E. Kim, M.J. Kim, J.S. Lee, J.K. Rho, S.Y. Jeong, E.K. Choi, C.S. Kim, J.J. Hwang, Vorinostat enhances gefitinibinduced cell death through reactive oxygen speciesdependent cleavage of HSP90 and its clients in nonsmall cell lung cancer with the EGFR mutation, Oncol Rep 41(1) (2019) 525-533.
[68] S. Takeuchi, T. Hase, S. Shimizu, M. Ando, A. Hata, H. Murakami, T. Kawakami, K. Nagase, K. Yoshimura, T. Fujiwara, A. Tanimoto, A. Nishiyama, S. Arai, K. Fukuda, N. Katakami, T. Takahashi, Y. Hasegawa, T.K. Ko, S.T. Ong, S. Yano, Phase I study of vorinostat with gefitinib in BIM deletion polymorphism/epidermal growth factor receptor mutation double-positive lung cancer, Cancer Sci 111(2) (2020) 561-570.
[69] A.A. Ahmed, S. Neidle, A G-Quadruplex-Binding Small Molecule and the HDAC Inhibitor SAHA (Vorinostat) Act Synergistically in Gemcitabine-Sensitive and Resistant Pancreatic Cancer Cells, Molecules 25(22) (2020).
[70] A.M. Molina, J.C. van der Mijn, P. Christos, J. Wright, C. Thomas, J.P. Dutcher, D.M. Nanus, S.T. Tagawa, L.J. Gudas, NCI 6896: a phase I trial of vorinostat (SAHA) and isotretinoin (13-cis retinoic acid) in the treatment of patients with advanced renal cell carcinoma, Invest New Drugs 38(5) (2020) 1383-1389.
[71] N. Pinto, S.G. DuBois, A. Marachelian, S.J. Diede, A. Taraseviciute, J.L. Glade Bender, D. Tsao-Wei, S.G. Groshen, J.M. Reid, D.A. Haas-Kogan, C.P. Reynolds, M.H. Kang, M.S. Irwin, M.E. Macy, J.G. Villablanca, K.K. Matthay, J.R. Park, Phase I study of vorinostat in combination with isotretinoin in patients with refractory/recurrent neuroblastoma: A new approaches to Neuroblastoma Therapy (NANT) trial, Pediatr Blood Cancer 65(7) (2018) e27023.
[72] T. Luu, K.P. Kim, S. Blanchard, B. Anyang, A. Hurria, L. Yang, J.H. Beumer, G. Somlo, Y. Yen, Phase IB trial of ixabepilone and vorinostat in metastatic breast cancer, Breast Cancer Res Treat 167(2) (2018) 469-478.
[73] R. Lan, Q. Wang, Deciphering structure, function and mechanism of lysine acetyltransferase HBO1 in protein acetylation, transcription regulation, DNA replication and its oncogenic properties in cancer, Cell Mol Life Sci 77(4) (2020) 637-649.
[74] G.J. Sung, S.H. Kim, S. Kwak, S.H. Park, J.H. Song, J.H. Jung, H. Kim, K.C. Choi, Inhibition of TFEB oligomerization by co-treatment of melatonin with vorinostat promotes the therapeutic sensitivity in glioblastoma and glioma stem cells, J Pineal Res 66(3) (2019) e12556.
[75] G. Zhao, J. Jia, L. Wang, Y. Zhang, H. Yang, Y. Lu, R. Yu, H. Liu, Y. Zhu, Local Delivery of Minocycline and Vorinostat Targets the Tumor Microenvironment to Inhibit the Recurrence of Glioma, Onco Targets Ther 13 (2020) 11397-11409.
[76] R. Ragheb, G. Venton, R. Chelbi, N. Bonnet, T. Le Treut, V. Ivanov, C. Mercier, P. Poulin, N. Beaufils, J. Gabert, P. Suchon, P. Rihet, B. Loriod, B. Kahn-Perles, R.T. Costello, Vorinostat and Mithramycin A in combination therapy as an interesting strategy for the treatment of Sezary T lymphoma: a transcriptomic approach, Arch Dermatol Res 309(8) (2017) 611-623.
[77] B. Liao, Y. Zhang, Q. Sun, P. Jiang, Vorinostat enhances the anticancer effect of oxaliplatin on hepatocellular carcinoma cells, Cancer Med 7(1) (2018) 196-207.
[78] S. Liu, K. Zhang, Q. Zhu, Q. Shen, Q. Zhang, J. Yu, Y. Chen, W. Lu, Synthesis and biological evaluation of paclitaxel and vorinostat co-prodrugs for overcoming drug resistance in cancer therapy in vitro, Bioorg Med Chem 27(7) (2019) 1405-1413.
[79] N.K. Chaturvedi, N.D. Hatch, G.L. Sutton, M. Kling, J.M. Vose, S.S. Joshi, A novel approach to eliminate therapy-resistant mantle cell lymphoma: synergistic effects of Vorinostat with Palbociclib, Leuk Lymphoma 60(5) (2019) 1214-1223.
[80] C.P. Rodriguez, Q.V. Wu, J. Voutsinas, J.R. Fromm, X. Jiang, V.G. Pillarisetty, S.M. Lee, R. Santana-Davila, B. Goulart, C.S. Baik, L.Q.M. Chow, K. Eaton, R. Martins, A Phase II Trial of Pembrolizumab and Vorinostat in Recurrent Metastatic Head and Neck Squamous Cell Carcinomas and Salivary Gland Cancer, Clin Cancer Res 26(4) (2020) 837-845.
[81] B. Jing, J. Jin, R. Xiang, M. Liu, L. Yang, Y. Tong, X. Xiao, H. Lei, W. Liu, H. Xu, J. Deng, L. Zhou, Y. Wu, Vorinostat and quinacrine have synergistic effects in T-cell acute lymphoblastic leukemia through reactive oxygen species increase and mitophagy inhibition, Cell Death Dis 9(6) (2018) 589.

[82] E. Galanis, S.K. Anderson, C.R. Miller, J.N. Sarkaria, K. Jaeckle, J.C. Buckner, K.L. Ligon, K.V. Ballman, D.F. Moore, Jr., M. Nebozhyn, A. Loboda, D. Schiff, M.S. Ahluwalia, E.Q. Lee, E.R. Gerstner, G.J. Lesser, M. Prados, S.A. Grossman, J. Cerhan, C. Giannini, P.Y. Wen, O. Alliance for Clinical Trials in, Abtc, Phase I/II trial of vorinostat combined with temozolomide and radiation therapy for newly diagnosed glioblastoma: results of Alliance N0874/ABTC 02, Neuro Oncol 20(4) (2018) 546-556.
[83] Q. Xu, C. Liu, J. Zang, S. Gao, C.J. Chou, Y. Zhang, Discovery of a Novel Hybrid of Vorinostat and Riluzole as a Potent Antitumor Agent, Front Cell Dev Biol 8 (2020) 454.
[84] D.O. Persky, H. Li, L.M. Rimsza, P.M. Barr, L.L. Popplewell, C.L. Bane, A. Von Gehr, M. LeBlanc, R.I. Fisher, S.M. Smith, J.W. Friedberg, A phase I/II trial of vorinostat (SAHA) in combination with rituximab-CHOP in patients with newly diagnosed advanced stage diffuse large B-cell lymphoma (DLBCL): SWOG S0806, Am J Hematol 93(4) (2018) 486-493.
[85] X. Hao, W. Xing, J. Yuan, Y. Wang, J. Bai, J. Bai, Y. Zhou, Cotargeting the JAK/STAT signaling pathway and histone deacetylase by ruxolitinib and vorinostat elicits synergistic effects against myeloproliferative neoplasms, Invest New Drugs 38(3) (2020) 610-620.
[86] X. Kou, Y. Yang, X. Jiang, H. Liu, F. Sun, X. Wang, L. Liu, H. Liu, Z. Lin, L. Jiang, Vorinostat and Simvastatin have synergistic effects on triple-negative breast cancer cells via abrogating Rab7 prenylation, Eur J Pharmacol 813 (2017) 161-171.
[87] S.W. Gordon, W.P. McGuire, 3rd, D.A. Shafer, R.K. Sterling, H.M. Lee, S.C. Matherly, J.D. Roberts, P. Bose, M.B. Tombes, E.E. Shrader, A.A. Ryan, M. Kmieciak, T. Nguyen, X. Deng, D. Bandyopadhyay, P. Dent, A.S. Poklepovic, Phase I Study of Sorafenib and Vorinostat in Advanced Hepatocellular Carcinoma, Am J Clin Oncol 42(8) (2019) 649-654.
[88] S. Bekeschus, A. Kramer, E. Suffredini, T. von Woedtke, V. Colombo, Gas Plasma Technology-An Asset to Healthcare During Viral Pandemics Such as the COVID-19 Crisis?, IEEE Trans Radiat Plasma Med Sci 4(4) (2020) 391-399.
[89] T. Jin, Y. Xu, C. Dai, X. Zhou, Q. Xu, Z. Wu, Cold atmospheric plasma: A non-negligible strategy for viral RNA inactivation to prevent SARS-CoV-2 environmental transmission, AIP Adv 11(8) (2021) 085019.
[90] Z. Chen, G. Garcia, Jr., V. Arumugaswami, R.E. Wirz, Cold atmospheric plasma for SARS-CoV-2 inactivation, Phys Fluids (1994) 32(11) (2020) 111702.
[91] G. Jungbauer, D. Moser, S. Muller, W. Pfister, A. Sculean, S. Eick, The Antimicrobial Effect of Cold Atmospheric Plasma against Dental Pathogens-A Systematic Review of In-Vitro Studies, Antibiotics (Basel) 10(2) (2021).
[92] F. Theinkom, L. Singer, F. Cieplik, S. Cantzler, H. Weilemann, M. Cantzler, K.A. Hiller, T. Maisch, J.L. Zimmermann, Antibacterial efficacy of cold atmospheric plasma against Enterococcus faecalis planktonic cultures and biofilms in vitro, PLoS One 14(11) (2019) e0223925.
[93] W.L. Hui, D. Ipe, V. Perrotti, A. Piattelli, Z. Fang, K. Ostrikov, A. Quaranta, Novel technique using cold atmospheric plasma coupled with air-polishing for the treatment of titanium discs grown with biofilm: An in-vitro study, Dent Mater 37(2) (2021) 359-369.
[94] J.Y. Lee, K.H. Kim, S.Y. Park, S.Y. Yoon, G.H. Kim, Y.M. Lee, I.C. Rhyu, Y.J. Seol, The bactericidal effect of an atmospheric-pressure plasma jet on Porphyromonas gingivalis biofilms on sandblasted and acid-etched titanium discs, J Periodontal Implant Sci 49(5) (2019) 319-329.
[95] Y. Yao, K. Song, H. Chen, X. Ding, Q. Shi, X. Lu, Y. Cao, In vitro and in vivo research of atmosphere pressure nonequilibrium plasmas on root canal disinfection: implication for alternative strategy for irrigation, Clin Oral Investig 25(10) (2021) 5833-5842.
[96] M. Kury, F. Moura Antonialli, S.S. LE, C. Pereira Machado Tabchoury, M. Giannini, F.L. Esteban Florez, V. Cavalli, Effects of violet radiation and nonthermal atmospheric plasma on the mineral contents of enamel during in-office dental bleaching, Photodiagnosis Photodyn Ther 31 (2020) 101848.
[97] B. Celik, I.D. Capar, F. Ibis, N. Erdilek, U.K. Ercan, Deionized water can substitute common bleaching agents for nonvital tooth bleaching when treated with non-thermal atmospheric plasma, J Oral Sci 61(1) (2019) 103-110.
[98] J.N. Stasic, J.K. Pficer, B. Milicic, N. Puac, V. Miletic, Effects of non-thermal atmospheric plasma on dentin wetting and adhesive bonding efficiency: Systematic review and meta-analysis, J Dent 112 (2021) 103765.
[99] X. Qi, X.M. Zhu, X. Liu, J. Li, L.X. Zhao, H.P. Li, J. Tan, Effects of a helium cold atmospheric plasma on bonding to artificial caries-affected dentin, Dent Mater J (2021).
[100] A. Stancampiano, D. Forgione, E. Simoncelli, R. Laurita, R. Tonini, M. Gherardi, V. Colombo, The Effect of Cold Atmospheric Plasma (CAP) Treatment at the Adhesive-Root Dentin Interface, J Adhes Dent 21(3) (2019) 229-237.
[101] M.M. Awad, F. Alhalabi, A. Alshehri, Z. Aljeaidi, A. Alrahlah, M. Ozcan, H.H. Hamama, Effect of Non-Thermal Atmospheric Plasma on Micro-Tensile Bond Strength at Adhesive/Dentin Interface: A Systematic Review, Materials (Basel) 14(4) (2021).
[102] A. Aleinik, A. Baikov, G. Dambaev, E. Semichev, P. Bushlanov, Liver Hemostasis by Using Cold Plasma, Surg Innov 24(3) (2017) 253-258.

[103] B. Stratmann, T.C. Costea, C. Nolte, J. Hiller, J. Schmidt, J. Reindel, K. Masur, W. Motz, J. Timm, W. Kerner, D. Tschoepe, Effect of Cold Atmospheric Plasma Therapy vs Standard Therapy Placebo on Wound Healing in Patients With Diabetic Foot Ulcers: A Randomized Clinical Trial, JAMA Netw Open 3(7) (2020) e2010411.
[104] Y. Miao, P. Han, D. Hua, R. Zhou, Z. Guan, Q. Lv, X. Dai, Cold atmospheric plasma increases IBRV titer in MDBK cells by orchestrating the host cell network, Virulence 12(1) (2021) 679-689.
